# A New Model-Free Index of Dynamic Cerebral Blood Flow Autoregulation

**DOI:** 10.1371/journal.pone.0108281

**Published:** 2014-10-14

**Authors:** Max Chacón, José Luis Jara, Ronney B. Panerai

**Affiliations:** 1 Departamento de Ingeniería Informática, Universidad de Santiago de Chile, Santiago, Chile; 2 Department of Cardiovascular Sciences, University of Leicester, Leicester, United Kingdom; Tokyo Metropolitan Institute of Medical Science, Japan

## Abstract

The classic dynamic autoregulatory index (ARI), proposed by Aaslid and Tiecks, is one of the most widely used methods to assess the efficiency of dynamic cerebral autoregulation. Although this index is often used in clinical research and is also included in some commercial equipment, it exhibits considerable intra-subject variability, and has the tendency to produce false positive results in clinical applications. An alternative index of dynamic cerebral autoregulation is proposed, which overcomes most of the limitations of the classic method and also has the advantage of being model-free. This new index uses two parameters that are obtained directly from the response signal of the cerebral blood flow velocity to a transient decrease in arterial blood pressure provoked by the sudden release of bilateral thigh cuffs, and a third parameter measuring the difference in slope of this response and the change in arterial blood pressure achieved. With the values of these parameters, a corresponding classic autoregulatory index value could be calculated by using a linear regression model built from theoretical curves generated with the Aaslid-Tiecks model. In 16 healthy subjects who underwent repeated thigh-cuff manoeuvres, the model-free approach exhibited significantly lower intra-subject variability, as measured by the unbiased coefficient of variation, than the classic autoregulatory index (*p* = 0.032) and the Rate of Return (*p*<0.001), another measure of cerebral autoregulation used for this type of systemic pressure stimulus, from 39.23%±41.91% and 55.31%±31.27%, respectively, to 15.98%±7.75%.

## Introduction

*Cerebral autoregulation* (CA) is the mechanism responsible for maintaining blood flow relatively constant in the brain, despite changes in the arterial blood pressure (ABP) in the range 60–150 mmHg [1]. Traditionally, CA was assessed by the steady-state relationship between mean ABP and mean cerebral blood flow. The introduction of transcranial Doppler ultrasound made possible to measure non-invasively beat-to-beat fluctuations of blood velocity in the vessels of the brain, which allowed the identification of transient autoregulatory responses, as blood flow velocity is a good approximation of blood flow under normal conditions [2]–[4]. These two approaches have been distinguished as the “static” and “dynamic” characterisations of CA respectively [5].

Several methods have been proposed to characterise the efficiency of the dynamic cerebral blood flow autoregulatory response [6]. One of the most widely used methods was proposed by Tiecks *et al.* [5], designed from the previous work of Aaslid *et al.* [7]. This method evaluates the changes observed in cerebral blood flow velocity (CBFV) in response to changes in ABP provoked by the sudden release of inflated bilateral thigh cuffs.

The main characteristic of the Aaslid-Tiecks method is the use of a single second-order model to describe the relationship of these two complex signals, the ABP signal as input and the CBFV signal as output. This family of models is expressed by a differential equation that is governed by three parameters, whose properties have been extensively studied in many different fields such as economics, chemistry, electrical and mechanical engineering, and automatic control. Using the model equation, Aaslid and Tiecks defined particular combinations for its parameters to grade autoregulatory responses in ten distinctive levels. By feeding the model with the actual ABP signal generated by a thigh-cuff manoeuvre, for each combination of parameters, ten possible CBFV template response curves can be generated. The efficiency of the autoregulatory response can then be quantified with an autoregulatory index (ARI) by fitting the actual CBFV response to one of these templates, obtaining an integer value of ARI ranging from 0 (absence of autoregulation) to 9 (best autoregulation).The Aaslid-Tiecks method was incorporated in at least one piece of commercial ultrasound equipment, which enabled its rapid diffusion and consolidation as the traditional method to assess dynamic CA. Indeed, just in the last year, several improvements of the method have been proposed for its use in clinical applications [8]–[11]. Moreover, alternative indices have normally been compared to the Aaslid-Tiecks ARI (e.g. the Rate of Regulation (RoR) [5], [12], the Mean Flow Index (Mx) [13] and the Multimodal Pressure-Flow method (MMPF) [12], [14]), as have been the use of the index with different ABP stimuli (e.g. spontaneous variations [15]–[16], the Valsalva manoeuvre [12], [16]–[17] and the sit-to-stand manoeuvre [18]).

However, the broad application of the Aaslid-Tiecks method, in both research and clinical work, is not matched by its precision and reproducibility as should be expected from clinical assessment tools. The measurement seems to be robust when mean ARI values can be obtained from the repeated application of several thigh-cuff manoeuvres in the same subject. Nonetheless, it has been reported that its variability increases significantly when the number of repetitions is limited and that it generates many *false positives* [19]–[20]. Worst still, the method indicated the complete absence of autoregulation or very low ARI values (≤2.0) in a considerable number of manoeuvres in healthy subjects. Furthermore, these difficulties seem not to be limited to the assessment of healthy subjects. ARI values obtained for patients with severe head-injury were analysed in [21], in which a great variation of the index could also be noted and there was an important number of these pathological cases with high ARI values, suggesting that the method might also be frequently generating false negatives.

With these problems in mind, a study of the parameters of the Aaslid-Tiecks model was conducted in [22], which argued that the numeric precision and generalisation power of the model can be both increased by expanding the possible values of each of its three parameters and the space for their combinations. This work showed that by extending just one of the parameters, it was possible to obtain better measurements, significantly reducing the intra-subject variability and producing no zero-ARI values for healthy subjects. A subsequent cerebrovascular reactivity study found that more reliable measurement tools could be obtained by introducing this unconstrained parameter into an autoregressive moving average model for continuously assessing autoregulation responses during transient hypocapnia and hypercapnia [23].

In the present work, we propose a *new algorithm* for measuring the efficiency of dynamic CA, which is independent of any particular model that relates ABP as the input signal and CBFV as the output signal. This new model-free autoregulation index (mfARI) is obtained by gauging three relatively independent parameters that represent the general behaviour of the CBFV response signal and how this behaviour compares with the one observed for the ABP signal. We study the variability and reliability of the proposed index in comparison to two CA assessment tools first designed for thigh-cuff manoeuvres, namely the standard Aaslid-Tiecks ARI [5] and the RoR [7].

## Materials and Methods

### Subjects

Data from sixteen volunteer subjects of mean age 31.8±8.5 years (range 23–51) were recruited. Subjects were excluded if they had a history of cardiovascular disease, migraine, epilepsy, hypertension, cerebral aneurysm, intracerebral bleeding, or other pre-existing neurological disorders. The study was approved by the Leicestershire Research Ethics Committee, and all subjects gave written informed consent. These data correspond to the same set used in [19].

### Measurements

CBFV was recorded from one middle cerebral artery using a Scimed QVL-120 transcranial Doppler system with a 2-MHz transducer. ABP was measured with a non-invasive blood pressure monitor Finapres 2300 Ohmeda. Recordings were made with subjects in the supine position with the head elevated to 30°.

Transient blood pressure drops were provoked using thigh-cuff manoeuvres. Each manoeuvre consisted of inflating two large bilateral thigh cuffs 20 mmHg above peak systolic ABP, as measured by the Finapres device, in all cases to ensure the occlusion of the circulation to the lower extremities, which was maintained for two minutes. After this time, the Velcro fastenings on the thigh cuffs were simultaneously and rapidly released. Each subject underwent six thigh-cuff manoeuvres, allowing an interval of 8 minutes between manoeuvres to permit ABP and CBFV to return to their baseline values.

The ABP and CBFV signals were sampled at a rate of 200 samples per second per channel. Both signals were filtered with an eight-order Butterworth low-pass filter with a cut-off of 20 Hz. The beginning of cardiac cycles were marked from the diastolic values in the ABP wave; mean ABP and mean CBFV signals were calculated for each cardiac cycle and then interpolated and re-sampled with a constant sample rate of 5 Hz. This protocol is further described in [19].

Three measures of autoregulation efficiency were obtained for each thigh-cuff manoeuvre, namely the classic Aaslid-Tiecks ARI, the RoR and the proposed mfARI.

### Classic Aaslid-Tiecks Method

It is important to notice that the procedure to estimate an ARI value followed by Mahony *et al.* [19] had some differences from the original method proposed by Aaslid and Tiecks.

Both approaches generated ten predicted CBFV template responses for the observed ABP stimulus by introducing the specific combination of the Aaslid-Tiecks model's parameters for the ten levels of ARI values defined from zero to nine. Both approaches compared the acquired CBFV response signal with these template responses and the closest match was selected. The difference though is that whilst Tiecks *et al.* [5] fitted the best template using a least-squares method, Mahony *et al.* based their estimates on a correlation coefficient procedure to avoid the need to select a particular value of critical closing pressure. This was followed by a parabolic interpolation to obtain non-integer values of ARI.

In the statistical analysis described below, we performed comparisons of mfARI with ARI using the values reported by Mahony *et al.*, but for the analysis of residuals we also reported a comparison adopting the original proposal of Tiecks *et al.*, using the same value of 12 mmHg for critical closing pressure which they suggested, in combination with the least squares, in order to have estimates that could be compared to the least-squares procedure adopted to estimate mfARI.

### Rate of Regulation

RoR was calculated as described in [7]. Initially, the baseline values are estimated as the mean value exhibited by each signal in the 4 s immediately before the time of thigh cuffs release. Then the signals are normalised by diving them by their baseline value. The time course of the Cerebrovascular Resistance (CVR) can then be determined by dividing the normalised ABP by the normalised CBFV signals, and the rate in which CVR changes during the interval from 1 to 3.6 s after the thigh cuffs release can be estimated. By dividing this rate by the magnitude of the ABP drop, calculated as the normalised mean ABP during the same interval, the Rate of Regulation is finally obtained.

### Proposed New Method

The proposed measurement system uses three parameters that can be conceptually separated in two types. Two parameters describe the autoregulatory response observed in the CBFV signal induced by a thigh-cuff manoeuvre. The third parameter relates the CBFV response to the drop in ABP provoked by the manoeuvre.

These parameters can then be transformed into a single *continuous* value, in the same range as the classic ARI, using a transformation process referred to as *standardisation*.

#### Parameters describing the CBFV response signal

Two parameters were devised to represent the CBFV signal observed in reaction to a sudden ABP perturbation. These parameters were inspired by a long-standing idea in the fields of system identification and automatic control: the Ziegler-Nichols Reaction Curve Method [24], which indicates that arbitrary-order systems can be roughly represented using only the two parameters of a first-order system, namely the system time constant (*τ*) and steady state gain (*k_S_*).

Moreover, a further simplification is proposed and the response of the system is characterised by two straight lines, rather than using the exponential equation that describe a first-order system. Thus a fixed-length horizontal line is used to describe the steady state attained by the system and a (normally) positive line is utilised to portray the transient response observed, from the minimum point in the CBFV signal after the release of the thigh cuffs up to the point the steady state begins. These relatively independent approximations allowed closer fittings of the response than attempting to adjust the specific curvature of a single first-order response. Moreover, following Occam's razor [25], two straight lines are the simplest possible description of the CBFV response.

**Figure 1:**
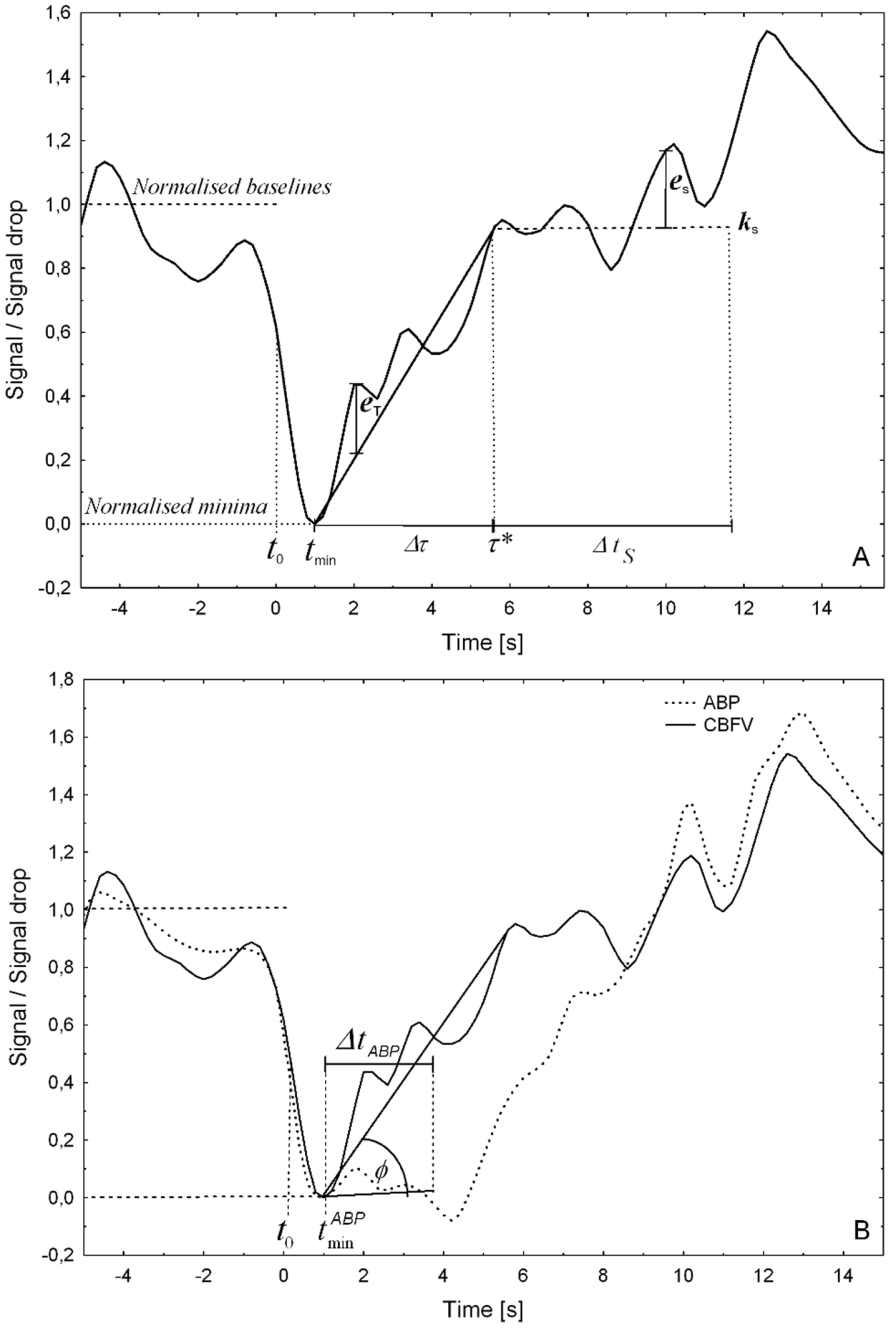
mfARI parameters in typical ABP and CBFV signals observed for a thigh-cuff manoeuvre. In the characterisation of the CBFV response (A), the signal is first normalised so that the baseline level equals unity and the minimum value equals zero; *t_0_* is the time of thigh cuffs release and *t_min_* is the time of minimum signal; τ* is the time at which the transient and stable responses produced the lowest errors; *Δτ* is the duration of the transient response and *Δτ_s_* is the duration of the stable state response considered in the optimisation procedure to search for τ*; the solid straight line is the representation of the transient response; the segmented straight line is the representation of the constant steady state response (*k_S_*). In the characterisation of the ABP stimulus (B), the signal (dotted line) is also normalised before the analysis; *t_0_* is as above and 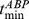 is the time of minimum ABP signal; *Δt_ABP_* is the duration of the segment of ABP signal considered in determining the slope of the straight line that represent the ABP recovery. The angle between the lines that represent the CBFV transient response and the ABP recovery signal corresponds to the *φ* parameter.

Fig. 1A shows a normalised CBFV response signal of one representative thigh-cuff manoeuvre of one subject. In this example, a transient response can clearly be seen for a short period of time (*Δτ*) immediately after the autoregulation system starts acting to recover a normal CBFV level. The initial time 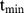 is defined as the time the signal exhibited its minimum value within a fixed-length time window after the release of the thigh cuffs (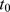), which in this study was set to 6 s (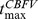 in Table S1). It should be noticed that CBFV responses were normalised by the amplitude of the drop produced by the release of the thigh cuffs, so that a change of one unit occurs between the baseline of the signal (i.e. the mean signal value up to 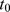) and its minimum value at time 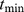. The signal of Fig. 1A also exhibits a clear steady state response, i.e. the output of the system in the long run, which in this proposal is considered to be a constant value.

A key part of the proposed method is to adequately determine *τ*, i.e. the *time point in which the CBFV transient response ends*. Firstly, it defines the first parameter: the time interval *Δτ*, as from *t_min_* to *τ*, both time points included. The straight line, usually with a positive slope, that best fit the portion of CBFV signal in this time interval can then be estimated to characterise the observed transient response. In addition, as the *τ* parameter defines the end of the transient response, it also marks the beginning of the steady state response. Consequently, it also defines *k_S_* as *CBFV*(*τ*). With this parameter, a constant straight line can be outlined to characterise the observed steady state response relative to the baseline.

An optimisation problem to search for the best value of *τ* was devised, which minimises the mean squared error (MSE) between the values of the CBFV response and the two lines that represent the behaviour of the signal during the transient and steady state responses respectively. Mathematically, the problem is formulated as:
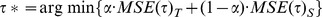
(1)

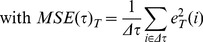
(2)

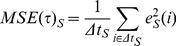
(3)

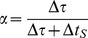
(4)in which each *e_T_*(*t*) and *e_S_*(*t*) is the difference between the sample value in the observed CBFV response and the sample value in the straight line that approximates it (at sampling time *t*) during the transient and steady state respectively. Thus, τ* is the resulting optimum time-point for which the MSE of the first *Δτ*+*Δt_S_* seconds of the CBFV signal, in regard to the lines that characterise the transient and steady state responses, is minimum. To solve the equation, it is sufficient to evaluate the formula for every possible value of *τ* in a discrete range [*τ _min_*, *τ _max_*], whose size depends on the sample rate used for the CBFV signal and the range of values obtained from templates generated with the classic model of Aaslid and Tiecks, as it is explained below.

#### Comparing the ABP and CBFV response signals

The autoregulatory efficiency is related to the ability of rapidly recovering a steady cerebral blood flow after a perturbation in the systemic blood pressure. Consequently, it is important to consider the *disassociation* of these two signals.

For this, the ideas of Novak *et al.* were considered, who have estimated the angle between the CBFV and ABP signals using non-stationary methods [13]–[14]. However, since only short time signals are evaluated in this work, they do not present stationarity problems, thus the angle that differentiate them can be directly measured from the normalised signals. For this, the straight line that best fits the segment of normalised ABP signal from the time it exhibits its minimum value and remains low (*Δt_ABP_* in table S1) is estimated to describe its behaviour. This minimal ABP signal value is sought in a time interval from *t*_0_ and *t*_0_+2 s (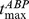 in Table S1) for the current study.

Thus, the third parameter of the new measurement system, named **ϕ**, corresponds to the angle observed between the straight line that characterises the CBFV transient response and the straight line that represents the return of the ABP signal, measured in degrees (Fig. 1B). More specifically, **ϕ** is the difference of the two angles observed between each straight line and the time axis, both limited to values between 0° and 90°. These angles are obtained as the arctangent of the slope of each straight line.

It must be noted that the ABP signal is also normalised as described above for the CBFV signal. Therefore, the slopes of the straight lines are measured in the same magnitude relative to the amplitude of the drop produced by the release of the thigh cuffs in the corresponding signal they represent.

#### Standardisation to ARI values

The new measurement system is not limited to an efficiency index in any particular scale. Nevertheless, it was decided to use the well-know scale defined by the classic Aaslid-Tiecks ARI, which range from zero to nine.

For this, the numeric resolution of the index was first increased by interpolating the ten original combinations of parameters with a cubic spline, in order to obtain 91 combinations, extending the precision of the ARI values to one decimal place.

91 theoretical responses were then generated using this extended set of combinations by applying a normalised negative ABP step stimulus. Using the method described above, the *Δτ*, *k_S_* and **ϕ** parameters were gauged from each theoretical response to characterise its behaviour. The lower and upper bounds used in calculating these parameters can be found in Table S1.

Supposing a linear relationship, it was possible to estimate the coefficients of a multivariate linear regression that estimated new continuous ARI values based on these parameters. The 91 theoretical responses also provided lower and upper bounds for each parameter to be applied on both real ABP stimuli and real CBFV responses. By providing the regression coefficients as part of our main results, other researchers will be able to calculate mfARI for their own data.

### Statistical analysis

The significance of the coefficients of the linear regression described above was evaluated with Student's t-tests, while a F-test was used to assess the reliability of the regression. The coefficient of determination *R^2^* was also calculated to assess the regression's goodness of fit.

mfARI values were obtained by applying the proposed method to the ABP and CBFV signals observed for each thigh-cuff manoeuvre. Classic ARI values for each manoeuvre were obtained from [19]. The probability distributions of the values obtained for each thigh-cuff manoeuvre with both indices were visually evaluated by using 20-bin histograms in the interval [0, 9]. Mean indices for each subject were computed and compared, and a Bland-Altman plot [26] was utilised to assess their agreement.

An analysis of residuals was conducted to evaluate the goodness of fit between the observed CBFV signals and the CBFV responses generated by the two ARI approaches. Specifically we compared the MSE between the straight lines generated by each pair of (*Δτ*, *k_S_*) parameters and the actual CBFV signals, which we then compared to the MSE between the best template selected by the classic method and each observed CBFV signal for the same period of time.

RoR values were determined for each thigh-cuff manoeuvres as described above. The intra-subject variabilities of the three CA assessment tools, namely the classic ARI, the proposed mfARI and the RoR, were compared in terms of their standard deviation normalised as a percentage of the mean, that is, their unbiased coefficient of variation (CoV) [27].

The reproducibility of the three methods was also studied by comparing their absolute reliability with the Standard Error of Measurement (SEM). This reliability measure was calculated from one-way ANOVA for repeated measures tables as explained in [28] and corrected for missing values as suggested in [29].

The Anderson-Darling test was used to assess the normality of distributions [30]. Subject mean ARI values were compared with paired Student's t-tests and one-way ANOVA with repeated measures was used to assess differences in CoV. Tuckey's Honest Statistical Difference was used as post-hoc analysis. Data were log-transformed when assumptions of normality or homoscedasticity were unsupported. In all tests, a value *p*<0.05 was considered significant.

## Results

**Figure 2:**
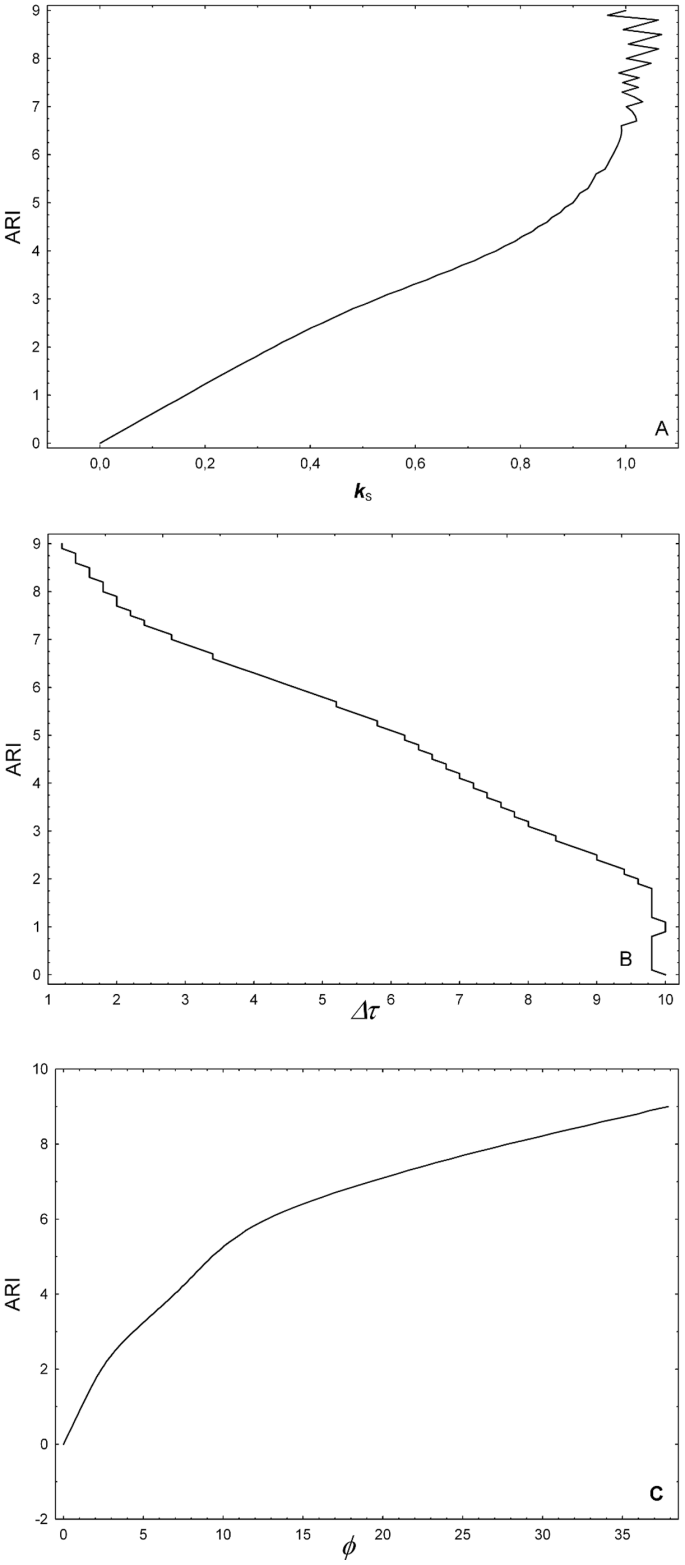
Standardisation of the mfARI parameters. By characterising 91 hypothetical responses, generated with the classic Aaslid-Tiecks model, for ARI values equally-spaced between 0.0 and 9.0, the range of possible values of the proposed parameters could be obtained: *k_S_* in A, *Δτ* in B and *φ* in C. All parameters exhibit a non-linear association with the ARI values. Although the behaviour of *k_S_* and *Δτ* values are similar to those observed for the original *K* and *T* parameters in the classic Aaslid-Tiecks model [22], the former are directly measured on the CBFV response signal, and not dependent on any particular model.

The values of the mfARI parameters, namely *Δτ*, *k_S_* and **ϕ**, obtained from the 91 theoretical step responses are plotted in Fig. 2. Table 1 contains the resulting standardisation regression model, which was found significant (*F*(3,87) = 13020.00, *p*<0.001) with a goodness of fit *R^2^* = 0.998. Student's t-tests for each parameter indicated that all of them significantly contributed to the model.

**Table 1:** Regression analysis for the standardisation procedure.

	Mean ± SD	Max.	Min.	Coefficient	t-test	*p*-value
*Intercept*				1.631	5.231	<0.001
*k_S_*	0.69±0.35	1,07	0	3.751	30.625	<0.001
*Δτ*	6.19±3.03 [s]	10.00 [s]	1.20 [s]	−0.137	−4.753	<0.001
*φ*	11.43°±10.56°	37.87°	0°	0.099	17.878	<0.001

Columns 1–3 present descriptive statistics for the values of the proposed parameters obtained from 91 theoretical step responses generated with the classic Aaslid-Tiecks model. Columns 4–6 report the coefficients resulting from the multivariate linear model built for the standardisation and their significance obtained from individual Student's t-tests for each parameter.

**Figure 3:**
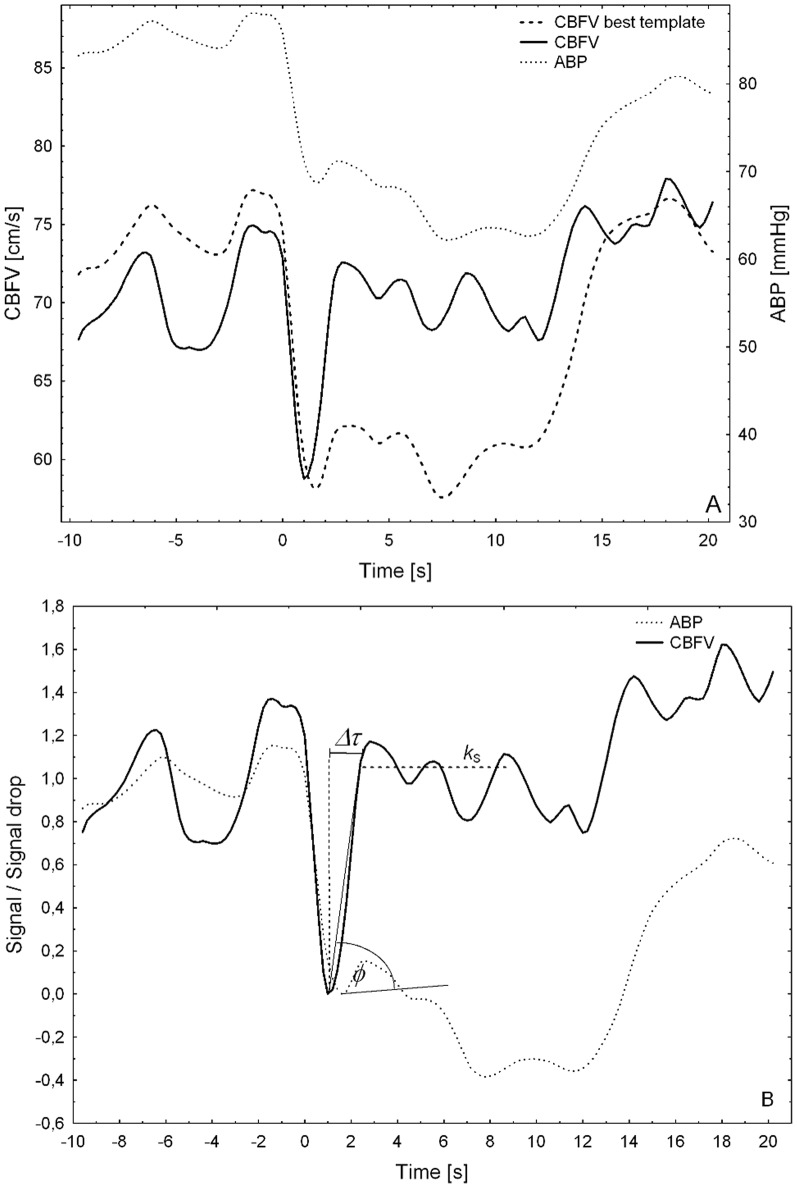
Calculation of the mfARI and classic ARI for one manoeuvre of one subject. Time-course of the ABP stimulus (dotted line) and the CBFV response (solid line) recorded in one of the thigh-cuff manoeuvres applied to one subject. In (A), the fitting of the closest template response (dashed line) with the classic method yielded an ARI value of 2.5. The plot on (B) presents the characterisation of the signals with the proposed system, which yielded an mfARI value of 8.5.

Six thigh-cuff manoeuvres were performed in each of the 16 subjects (96 in total), out of which seven (four subjects) presented unacceptable levels of noise in the recorded signals, and were discarded. Thus, a total of 89 manoeuvres were considered for the analysis. Fig. 3 shows the application of the proposed method to one case in which there is an important difference between the ARI value estimated with (A) the classic and (B) the model-free methods. Table 2 shows the mfARI values estimated for each manoeuvre.

**Table 2:** New ARI values.

	Test iteration						
Subject	1	2	3	4	5	6	Mean ± SD
1	3.9	5.1	3.0	4.6	3.7	4.6	4.1±0.76
2	6.5	5.2	4.8	6.1	5.4	5.9	5.7±0.63
3			5.4	4.5	5.5	5.6	5.2±0.51
4	5.1	5.9	5.9	7.6	5.4	4.9	5.8±0.97
5	5.2	4.8	4.0	4.9	5.5	3.7	4.7±0.70
6	3.5	5.7	5.2	5.7	5.0	2.7	4.6±1.25
7		4.9	6.1	6.4	4.3	9.0	6.1±1.81
8	5.8	5.6	5.6	3.8	6.3	6.2	5.5±0.91
9	4.3	7.6	4.0	6.4	5.3	7.9	5.9±1.65
10	5.2		5.4	5.2	6.4	6.0	5.6±0.54
11	7.3	7.6	6.3	5.0	6.0	8.1	6.7±1.15
12			7.0	6.2	6.2		6.5±0.46
13	4.6	4.8	4.9	4.8	4.9	5.3	4.9±0.23
14	6.6	7.4	7.1	5.9	6.5	7.6	6.8±0.63
15	4.5	4.1	4.5	3.5	4.4	3.5	4.1±0.48
16	8.5	5.7	4.6	6.1	8.5	7.7	6.8±1.62

mfARI values estimated for each thigh-cuff manoeuvre analysed and mean ± SD by subject.

The residual analysis indicated that the proposed method yielded an average MSE of 4.50±5.71 cm/s, which resulted much lower than the 40.35±41.52 cm/s obtained by the best templates selected by the classic approach as in [19]. When template responses were fitted minimising error, the original Aaslid-Tieck approach yielded an average MSE of 32.28±32.94 cm/s.

**Figure 4:**
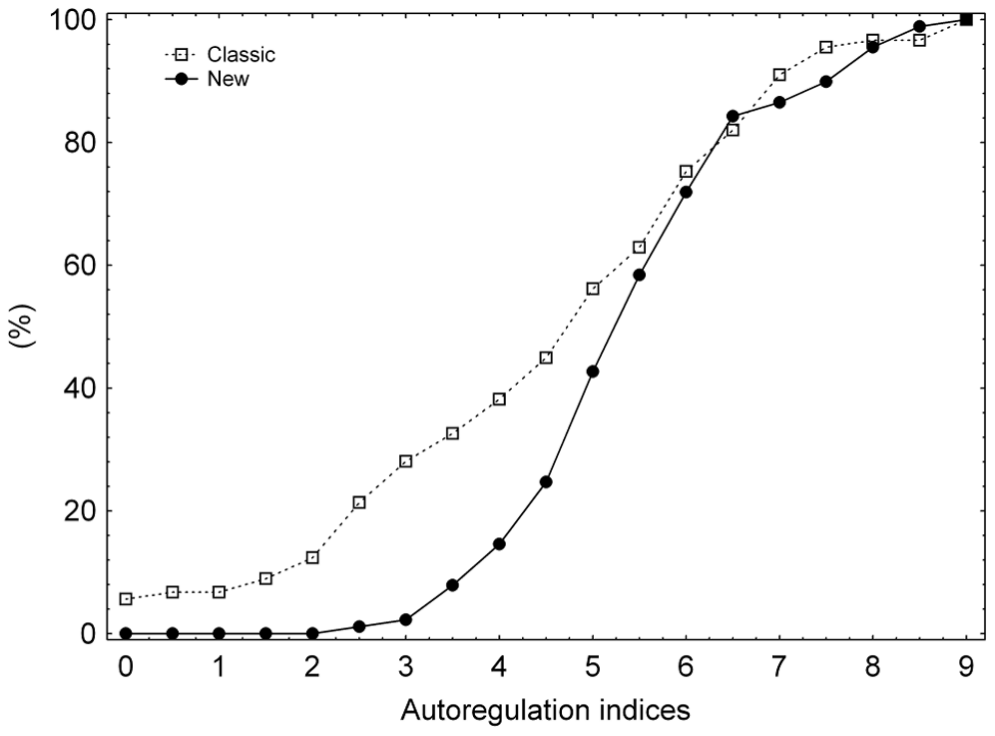
Comparison of the cumulative distributions of both indices. Percentage cumulative probability distribution of mfARI values (filled circles, solid line) compared to the corresponding distribution for classic ARI values (open squares, dotted line).

mfARI values for manoeuvres showed no evidence of deviation from normality (*A* = 0.588, *p* = 0.122), as did classic ARI values (*A* = 0.687, *p* = 0.071). Fig. 4 presents the cumulative distributions of both indices, in which it can be seen that the classic method assigned very low ARI values (≤2.0) to several thigh-cuff manoeuvres, whereas the lowest mfARI value assigned was 2.7 (for the sixth manoeuvre of subject 6, which obtained a classic ARI value of 0.0).

**Figure 5:**
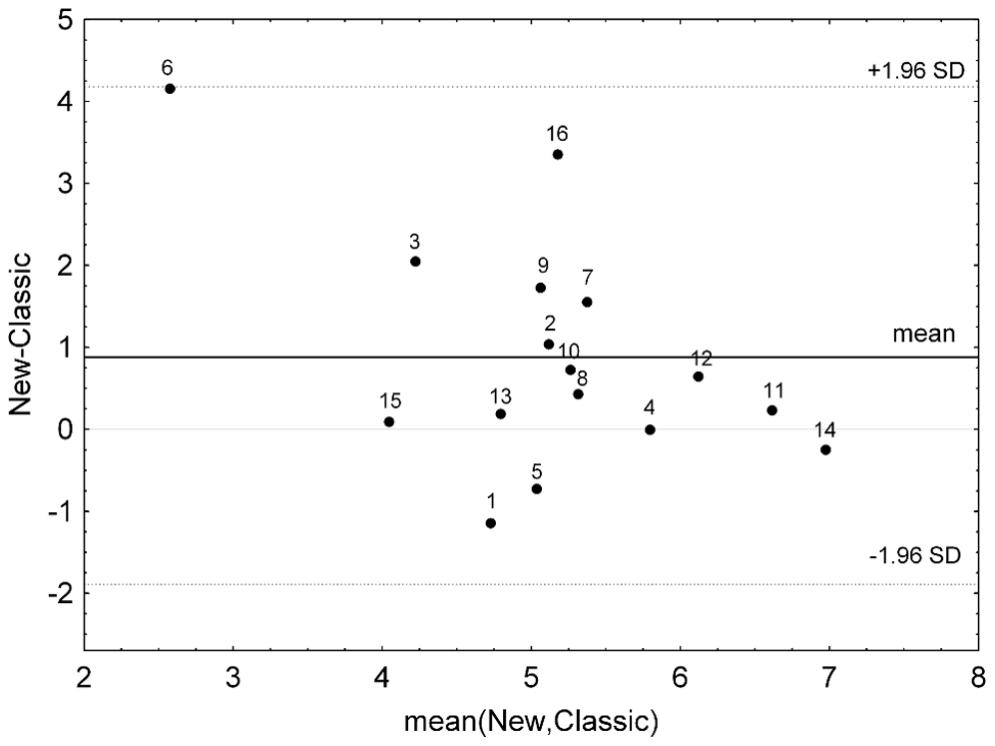
Bland-Altman plot of mean values for each subject. The difference between the new mfARI and classic ARI indices shows the bias (solid dark line) and the 95% confidence interval (dotted line) indicates the limits of agreement.

Table 2 also shows the Mean ± SD mfARI values by each subject. Fig. 5 depicts the agreement of these values and the subject mean ARI reported in [19]. Both sets of values did not contradict the assumption of being normally distributed (mfARI: *A* = 0.262, *p* = 0.656; classic ARI: *A* = 0.432, *p* = 0.267). Subject mean mfARI values were higher than classic ARI values in general, especially for the subjects that obtained very low ARI values. The population mean mfARI (5.6±0.90) resulted significantly higher (*t*(15) = 2.47, *p*<0.026) than the one reported for the classic method (4.7±1.50).

**Figure 6:**
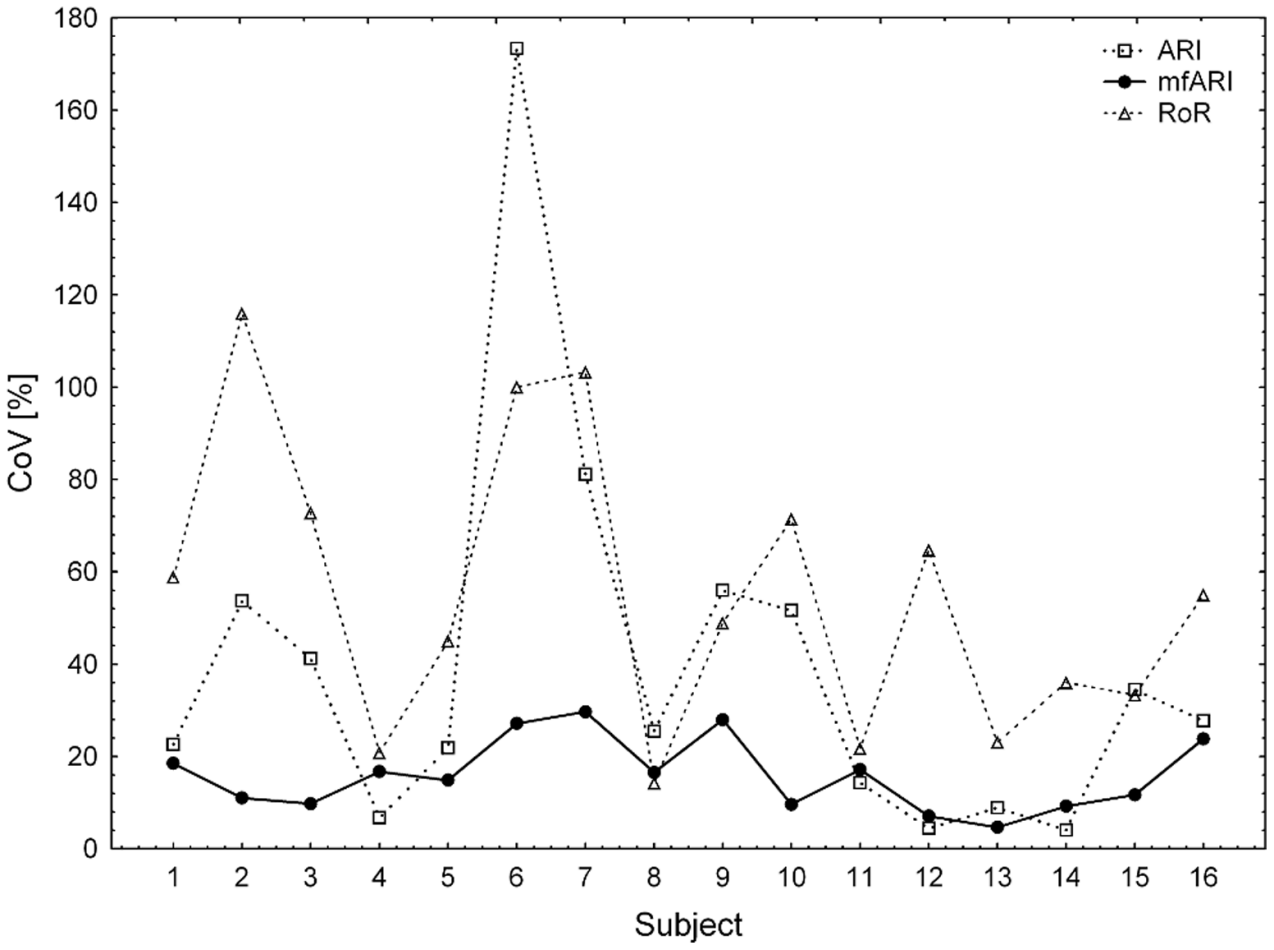
Intra-subject variability. Subject mean CoV values obtained with the classic ARI (open squares, dotted line), the mfARI (filled circles, solid line) and the RoR (open triangles, dashed line).

Fig. 6 shows the distributions of mean CoV across subjects, which did not present signs of deviation from normality for mfARI (*A* = 0.433, *p* = 0.265) and RoR (*A* = 0.350, *p* = 0.426), in contrast to the case of the classic ARI (*A* = 1.253, *p* = 0.002). The mfARI exhibited a population mean CoV of 15.98%±7.75%, which was significant lower than the mean CoV shown by both the classic ARI (39.23%±41.91%, *p* = 0.032) and the RoR (55.31%±31.27%, *p*<0.001). P-values were estimated with log-transformed data.

The absolute reliability of mfARI (SEM = 1.028 or 18.53%) resulted better than for both the classic ARI (SEM = 1.657 or 35.36%) and the RoR (SEM = 0.187 or 78.11%).

## Discussion

The proposed method is mainly based on two concepts: firstly, CA can be assessed by characterising the CBFV response to an external ABP stimulus and the relationship stimulus-response; and secondly, that this characterisation must be as simple as possible. In the current proposal, signals were characterised by fitting straight lines, which can be represented with three parameters. These parameters might then be used as the independent variables in a regression model that would allow the standardisation of the measure to a specific range of values. In this study, a linear regression with the values of the classic ARI was considered.

The linear regression obtained warrants the *conceptual* equivalence of the mfARI and the classic ARI. Any difference between the values estimated for the same thigh-cuff manoeuvre will be due to the quality of the fitting process allowed by their parameters. Also, mfARI values are not limited to integer numbers as in the original approach [5], nor are the result of the interpolation between integer values as in [19].

Although the standardisation procedure bounded the range of the mfARI, these limitations are different from the ones applying to the classic ARI. The latter is morphologically constrained by a small set of possible values for the generating parameters of its differential equation, which restricts its ability to fit the wide spectrum of real autoregulatory responses. In contrast, mfARI could retain all possible combinations of its parameters and, despite the fact that the bounds for their values were estimated from theoretical responses, it was able to produce better fittings for real responses.

Mean mfARI values resulted higher (0.90 units in average) for the healthy subjects studied. Whilst the classic method seldom produced subject mean ARI values over 6.5, subject mean mfARI within this range were more frequently seen, making a better use of the full range of values defined from zero to nine. In our view, this is an important improvement over the classic ARI that has been criticised for generating low ARI values for healthy subjects. The difficulties of the classic method to produce higher ARI values might be due to the particularities of the underlying second-order system, which requires the occurrence of decaying oscillations (*under-dumping*) in the CBFV signal to generate them, which are not easily found in real responses. In contrast, the proposed method captured appropriate combinations of mfARI parameters that fitted more closely good autoregulatory responses. For example, in the first manoeuvre of subject 16, there was a good autoregulatory response (Fig. 3). This was captured by the new index (mfARI = 8.5) but missed by the classic one (ARI = 2.5). Moreover, the classic method produced very low values (ARI≤2.0) for several thigh-cuff manoeuvres (over 10%). In contrast, the proposed method assigned mfARI values in the range 2.7–7.9 (5.0±1.60) to these cases.

The mfARI also showed a reduction in intra-subject variability with a population mean CoV reduction of 59.27% in relation to the classic ARI and of 71.11% in relation to the RoR. This is also an advancement of the proposed method over the classic ARI and RoR, as limited variations of the index is expected for a group of healthy subjects. This reduction in variability explains the superior absolute reliability exhibited by mfARI, with reductions in SEM of 47.60% when compared to the classic ARI and 76.28% when compared to the RoR.

In summary, mfARI has shown simultaneously a reduction in both intra- and inter-subject variability when applied to CA responses on healthy subjects, improving both the precision and reproducibility of the measure with respect to the classic ARI and the RoR. Moreover, the method offers advantages in clinical application: both ABP and CBFV signals can be recorded with non-invasive equipment that is usually available in health settings, and the autoregulatory response underlying in these signals can be characterised with three computationally-inexpensive parameters. These features could initially contribute to reducing the number of thigh-cuff manoeuvres necessary to assess the autoregulatory response of patients. The proposed method could also easily be adapted to work with other techniques to produce sudden changes in ABP, such as sit-to-stand [18], [31] or Valsalva [17] manoeuvres. Furthermore, as with the classic ARI, mfARI could be used in combination with subject-specific CA models to remove the need of ABP stimuli, extending the application of the index to patients for which changes in intracranial pressure could be potentially harmful. Linear models, such as transfer functions [32] and autoregressive models [33]–[34], could be built from recordings of spontaneous variation of ABP and used to assess CA through the model's step response. Additionally, a more general approach could be obtained by measuring responses from non-linear models, similar to the one presented in [35].

The limitations of assessing dynamic CA with thigh-cuff manoeuvres and transcranial Doppler ultrasound have been extensively discussed [5], [7], [16], [19], [36], and are mainly related to three aspects. Firstly, despite the manoeuvre being widely undertaken on patients, the inflation of thigh cuffs up to 20 mmHg above systolic ABP causes moderate pain. However, this was not reported as a problem in the work of Mahony *et al.*, from which the subjects' data for this study were taken. Secondly, the insonation of any cerebral artery is achieved through the so called *acoustic window* in the skull, which is not present in every subject. This was also not reported as an issue for the data used here. Finally, transcranial Doppler ultrasound does not measure cerebral blood flow directly, which can only be considered comparable to the measured CBFV if the cross-sectional areas of the insonated cerebral arteries remained constant during the assessment.

It could also be argued that the characterisation of the CBFV response using the parameters of a linear first-order system could be limiting the proposed approach, as it is simpler than the original second-order system put forth by Aaslid and Tiecks. However, the first-order model in the proposal plays a very different role than the second-order model in the classic approach. There is no suggestion that CA can be modelled as a first-order system. Rather the proposition is that, as in the Ziegler-Nichols method, the parameters of a first-order system can be used to characterise the responses observed for an unknown arbitrary-order system.

In this study, mfARI has been compared with measurement tools that were initially designed for thigh-cuff manoeuvres, namely the classic ARI and the RoR. To compare the proposed index against other methods to assess the efficiency of dynamic CA, such as the Mx index [13] and the MMPF method [12]–[14], it would be necessary to define new experimental settings aimed at recording ABP and CBFV signals from individuals subjected to different conditions than the ones considered in this study (e.g. under spontaneous blood pressure variations or performing Valsalva manoeuvres). Therefore, future work must be conducted to address these comparisons.

The recently proposed MRARI to assess CA from magnetic resonance images [37] is based on the same principles as the classic ARI, fitting exponential templates, though it uses a simplified model that consider only the CBFV responses, as the subject's ABP signal cannot be measured in the scanner. As two out of the three parameters of mfARI are derived from these signals, the method could be readily adapted to be used with magnetic resonance images to obtain local assessments of dynamic CA. Neither the RoR nor the Mx index nor the MMPF methods share this potential.

This initial study has inspected exclusively data from healthy subjects. Further research is also needed to assess the effectiveness of mfARI to distinguish healthy individuals from patients with impaired autoregulation. This could be achieved by evaluating the proposed index with data from healthy subjects in conditions that temporarily change their autoregulatory ability, such as breathing a mixture of air and CO_2_ [38] or re-breathing [39], as well as with data from patients affected by one of the pathologies that are known to impair the autoregulation mechanism.

## Supporting Information

Table S1 **Criteria when measuring the mfARI parameters.** The estimation of the three proposed parameters, namely *Δτ*, *k_S_* and **ϕ**, required the definition of certain criteria. Most of them were defined following the common practices in studies of dynamic cerebral autoregulation with thigh-cuff manoeuvres (for example [5], [7]). Others were determined by the extreme values observed in the 91 theoretical step responses generated with the classic Aaslid-Tiecks model.
(DOCX)
