# Supplementary material for: A New Model-Free Index of Dynamic Cerebral Blood Flow Autoregulation
Source: PLoS One. 2014 Oct 14;9(10):e108281. doi: 10.1371/journal.pone.0108281 (PMC4196773; doi:10.1371/journal.pone.0108281)
Supplement: Table S1 — Criteria when measuring the mfARI parameters. The estimation of the three proposed parameters, namely Δτ, kS and ϕ, required the definition of certain criteria. Most of them were defined following the common practices in studies of dynamic cerebral autoregulation with thigh-cuff manoeuvres (for example [5], [7]). Others were determined by the extreme values observed in the 91 theoretical step responses generated with the classic Aaslid-Tiecks model. (DOCX) [file pone.0108281.s001.docx]

| **Notation** | **Description** | **Value** |
| --- | --- | --- |
| *t_0_* | Time at which the thigh cuffs are released. | zero |
| $t_{\max}^{ABP}$ | Maximum time after *t_0_* to look for the minimum ABP signal. | 2 [s] |
| $t_{\min}^{ABP}$ | The time point at which the minimum ABP value is observed between [*t_0_* - $t_{\max}^{ABP}$]. | searched |
| *Δt_ABP_* | Duration of the segment of ABP signal from, and including, $t_{\min}^{ABP}$  to estimate the slope of the straight line that represent the recovery of ABP. | 6 [s] |
| $t_{\max}^{CBFV}$ | Maximum time after *t_0_* to look for the minimum CBFV signal. | 6 [s] |
| $t_{\min}$ | The time point at which the minimum CBFV value is observed between [*t_0_* - $t_{\max}^{CBFV}$]. | searched |
| *Δt_S_* | Duration of the segment of the CBFV steady state response considered in the optimisation of the duration of the CBFV transient response. | 6 [s] |
| *τ_min_* | Minimum possible value for the parameter *τ* from *t_min_*; it corresponds to the duration of the transient response in the theoretical step response with ARI=9.0. | 1.2 [s] |
| *τ_max_* | Maximum possible value for the parameter *τ* from *t_min_*; the selected value considers the duration of the transient response in the theoretical step response with ARI=0.1. | 10 [s] |
| *Δt_BASE_* | Duration of the segment immediately before *t_0_*, but including this point, used to estimate the baselines of both ABP and CBFV. | 10 [s] |
